# Supplementary material for: Comparison of post-discharge mortality and medical expenditures in COVID-19 patients according to mechanical ventilation and extracorporeal membrane oxygenation use: The LIFE study
Source: PLoS One. 2026 Mar 26;21(3):e0345939. doi: 10.1371/journal.pone.0345939 (PMC13020807; doi:10.1371/journal.pone.0345939)
Supplement: S1 File — Cox Regression Analysis of 180-Day Post-Discharge Mortality with Age Groups. Concordance = 0.718 (standard error = 0.011). CI, confidence interval; ECMO, extracorporeal membrane oxygenation; LOS, length of stay; MV, mechanical ventilation. S2 Table. Generalized Linear Model Analysis of 180-Day Post-Discharge Total Medical Expenditures with Age Groups. CI, confidence interval; ECMO, extracorporeal membrane oxygenation; LOS, length of stay; MV, mechanical ventilation. S3 Table. Cox Regression Analysis of 180-Day Post-Discharge Mortality with Charlson Comorbidity Index Scores. Concordance = 0.70 (standard error = 0.012). CI, confidence interval; ECMO, extracorporeal membrane oxygenation; LOS, length of stay; MV, mechanical ventilation. S4 Table. Cox Regression Analysis of 180-Day Post-Discharge Mortality with Elixhauser Comorbidity Index Scores. Concordance = 0.70 (standard error = 0.012). CI, confidence interval; ECMO, extracorporeal membrane oxygenation; LOS, length of stay; MV, mechanical ventilation. S5 Table. Generalized Linear Model Analysis of 180-Day Post-Discharge Total Medical Expenditures with Charlson Comorbidity Index Scores. CI, confidence interval; ECMO, extracorporeal membrane oxygenation; LOS, length of stay; MV, mechanical ventilation. S6 Table. Generalized Linear Model Analysis of 180-Day Post-Discharge Total Medical Expenditures with Elixhauser Comorbidity Index Scores. CI, confidence interval; ECMO, extracorporeal membrane oxygenation; LOS, length of stay; MV, mechanical ventilation. S7 Table. Cox Regression Analysis of 180-Day Post-Discharge Mortality with COVID-19 Variant Periods. Concordance = 0.718 (standard error = 0.011). CI, confidence interval; ECMO, extracorporeal membrane oxygenation; LOS, length of stay; MV, mechanical ventilation. S8 Table. Generalized Linear Model Analysis of 180-Day Post-Discharge Total Medical Expenditures with COVID-19 Variant Periods. CI, confidence interval; ECMO, extracorporeal membrane oxygenation; LOS, length o [file pone.0345939.s001.zip › Supporting Information/S5 Table.docx]

**S5 Table. Generalized Linear Model Analysis of 180-Day Post-Discharge Total Medical Expenditures with Charlson Comorbidity Index Scores**.

| **Independent Variables** | **Exp(β)** | **95% CI** | ***p*-value** |
| --- | --- | --- | --- |
| MV/ECMO (ref: Non-MV/ECMO) | 1.45 | 1.25–1.68 | <0.001 |
| Age | 1.01 | 1.01–1.02 | <0.001 |
| Male (ref: female) | 1.06 | 0.97–1.16 | 0.21 |
| Obesity | 0.60 | 0.41–0.93 | 0.015 |
| LOS | 1.01 | 1.01–1.01 | <0.001 |
| Hospitalization expenditure | 1.00 | 1.00–1.00 | 0.80 |
| Delirium on admission | 1.04 | 0.89–1.23 | 0.62 |
| Charlson score | 1.12 | 1.08–1.16 | <0.001 |

CI, confidence interval; ECMO, extracorporeal membrane oxygenation; LOS, length of stay; MV, mechanical ventilation.
