# Supplementary material for: Comparison of post-discharge mortality and medical expenditures in COVID-19 patients according to mechanical ventilation and extracorporeal membrane oxygenation use: The LIFE study
Source: PLoS One. 2026 Mar 26;21(3):e0345939. doi: 10.1371/journal.pone.0345939 (PMC13020807; doi:10.1371/journal.pone.0345939)
Supplement: S1 File — Cox Regression Analysis of 180-Day Post-Discharge Mortality with Age Groups. Concordance = 0.718 (standard error = 0.011). CI, confidence interval; ECMO, extracorporeal membrane oxygenation; LOS, length of stay; MV, mechanical ventilation. S2 Table. Generalized Linear Model Analysis of 180-Day Post-Discharge Total Medical Expenditures with Age Groups. CI, confidence interval; ECMO, extracorporeal membrane oxygenation; LOS, length of stay; MV, mechanical ventilation. S3 Table. Cox Regression Analysis of 180-Day Post-Discharge Mortality with Charlson Comorbidity Index Scores. Concordance = 0.70 (standard error = 0.012). CI, confidence interval; ECMO, extracorporeal membrane oxygenation; LOS, length of stay; MV, mechanical ventilation. S4 Table. Cox Regression Analysis of 180-Day Post-Discharge Mortality with Elixhauser Comorbidity Index Scores. Concordance = 0.70 (standard error = 0.012). CI, confidence interval; ECMO, extracorporeal membrane oxygenation; LOS, length of stay; MV, mechanical ventilation. S5 Table. Generalized Linear Model Analysis of 180-Day Post-Discharge Total Medical Expenditures with Charlson Comorbidity Index Scores. CI, confidence interval; ECMO, extracorporeal membrane oxygenation; LOS, length of stay; MV, mechanical ventilation. S6 Table. Generalized Linear Model Analysis of 180-Day Post-Discharge Total Medical Expenditures with Elixhauser Comorbidity Index Scores. CI, confidence interval; ECMO, extracorporeal membrane oxygenation; LOS, length of stay; MV, mechanical ventilation. S7 Table. Cox Regression Analysis of 180-Day Post-Discharge Mortality with COVID-19 Variant Periods. Concordance = 0.718 (standard error = 0.011). CI, confidence interval; ECMO, extracorporeal membrane oxygenation; LOS, length of stay; MV, mechanical ventilation. S8 Table. Generalized Linear Model Analysis of 180-Day Post-Discharge Total Medical Expenditures with COVID-19 Variant Periods. CI, confidence interval; ECMO, extracorporeal membrane oxygenation; LOS, length o [file pone.0345939.s001.zip › Supporting Information/S7 Table.docx]

**S7 Table. Cox Regression Analysis of 180-Day Post-Discharge Mortality with COVID-19 Variant Periods**.

| **Independent Variables** | **Hazard Ratio** | **95% CI** | ***p*-value** |
| --- | --- | --- | --- |
| MV/ECMO (ref: Non-MV/ECMO) | 1.67 | 1.26–2.20 | 0.003 |
| Delta variant period (ref: Pre-Delta variant period) | 0.88 | 0.66–1.17 | 0.38 |
| Age | 1.06 | 1.05–1.07 | <0.001 |
| Male (ref: female) | 1.24 | 1.03–1.50 | 0.027 |
| Obesity | 0.84 | 0.27–2.63 | 0.76 |
| LOS | 1.01 | 1.01–1.02 | <0.001 |
| Hospitalization expenditure | 1.00 | 1.00–1.00 | 0.025 |
| Delirium on admission | 0.96 | 0.71–1.29 | 0.76 |
| Hypertension | 0.78 | 0.64–0.96 | 0.017 |
| Diabetes | 1.13 | 0.91–1.41 | 0.26 |
| Lower respiratory disease | 1.43 | 1.06–1.92 | 0.019 |
| Heart disease | 1.01 | 0.83–1.23 | 0.94 |
| Kidney disease | 1.35 | 0.93–1.95 | 0.11 |
| Cerebrovascular disease | 1.00 | 0.80–1.25 | 0.98 |
| Dementia | 1.48 | 1.10–1.99 | 0.01 |
| Cancer | 1.93 | 1.57–2.37 | <0.001 |
| Liver disease | 1.92 | 1.19–3.11 | 0.008 |
| Delta variant period * MV/ECMO | 0.93 | 0.42–2.04 | 0.85 |
| Concordance = 0.718 (standard error = 0.011). CI, confidence interval; ECMO, extracorporeal membrane oxygenation; LOS, length of stay; MV, mechanical ventilation. | | | |
