# Supplementary material for: Comparison of post-discharge mortality and medical expenditures in COVID-19 patients according to mechanical ventilation and extracorporeal membrane oxygenation use: The LIFE study
Source: PLoS One. 2026 Mar 26;21(3):e0345939. doi: 10.1371/journal.pone.0345939 (PMC13020807; doi:10.1371/journal.pone.0345939)
Supplement: S1 File — Cox Regression Analysis of 180-Day Post-Discharge Mortality with Age Groups. Concordance = 0.718 (standard error = 0.011). CI, confidence interval; ECMO, extracorporeal membrane oxygenation; LOS, length of stay; MV, mechanical ventilation. S2 Table. Generalized Linear Model Analysis of 180-Day Post-Discharge Total Medical Expenditures with Age Groups. CI, confidence interval; ECMO, extracorporeal membrane oxygenation; LOS, length of stay; MV, mechanical ventilation. S3 Table. Cox Regression Analysis of 180-Day Post-Discharge Mortality with Charlson Comorbidity Index Scores. Concordance = 0.70 (standard error = 0.012). CI, confidence interval; ECMO, extracorporeal membrane oxygenation; LOS, length of stay; MV, mechanical ventilation. S4 Table. Cox Regression Analysis of 180-Day Post-Discharge Mortality with Elixhauser Comorbidity Index Scores. Concordance = 0.70 (standard error = 0.012). CI, confidence interval; ECMO, extracorporeal membrane oxygenation; LOS, length of stay; MV, mechanical ventilation. S5 Table. Generalized Linear Model Analysis of 180-Day Post-Discharge Total Medical Expenditures with Charlson Comorbidity Index Scores. CI, confidence interval; ECMO, extracorporeal membrane oxygenation; LOS, length of stay; MV, mechanical ventilation. S6 Table. Generalized Linear Model Analysis of 180-Day Post-Discharge Total Medical Expenditures with Elixhauser Comorbidity Index Scores. CI, confidence interval; ECMO, extracorporeal membrane oxygenation; LOS, length of stay; MV, mechanical ventilation. S7 Table. Cox Regression Analysis of 180-Day Post-Discharge Mortality with COVID-19 Variant Periods. Concordance = 0.718 (standard error = 0.011). CI, confidence interval; ECMO, extracorporeal membrane oxygenation; LOS, length of stay; MV, mechanical ventilation. S8 Table. Generalized Linear Model Analysis of 180-Day Post-Discharge Total Medical Expenditures with COVID-19 Variant Periods. CI, confidence interval; ECMO, extracorporeal membrane oxygenation; LOS, length o [file pone.0345939.s001.zip › Supporting Information/S2 Table.docx]

**S2 Table. Generalized Linear Model Analysis of 180-Day Post-Discharge Total Medical Expenditures with Age Groups.**

| **Independent Variables** | **Exp(β)** | **95% CI** | ***p*-value** |
| --- | --- | --- | --- |
| MV/ECMO (ref: Non-MV/ECMO) | 1.45 | 1.26–1.68 | <0.001 |
| Age, 65–74 years (ref: <65 years)  Age, ≥75 years (ref: <65 years) | 1.37  1.53 | 1.18–1.58  1.34–1.74 | <0.001  <0.001 |
| Male (ref: female) | 1.02 | 0.93–1.12 | 0.65 |
| Obesity | 0.63 | 0.43–0.97 | 0.025 |
| LOS | 1.01 | 1.01–1.01 | <0.001 |
| Hospitalization expenditure | 1.00 | 1.00–1.00 | 0.34 |
| Delirium on admission | 1.10 | 0.94–1.30 | 0.24 |
| Hypertension | 1.04 | 0.94–1.15 | 0.42 |
| Diabetes | 1.03 | 0.92–1.15 | 0.62 |
| Lower respiratory disease | 1.18 | 0.10–1.41 | 0.06 |
| Heart disease | 1.02 | 0.93–1.13 | 0.65 |
| Kidney disease | 1.62 | 1.31–2.03 | <0.001 |
| Cerebrovascular disease | 1.77 | 1.56–1.95 | <0.001 |
| Dementia | 1.21 | 1.00–1.47 | 0.06 |
| Cancer | 1.28 | 1.14–1.43 | <0.001 |
| Liver disease | 1.26 | 0.93–1.76 | 0.16 |

CI, confidence interval; ECMO, extracorporeal membrane oxygenation; LOS, length of stay; MV, mechanical ventilation.
