# Supplementary material for: Comparison of post-discharge mortality and medical expenditures in COVID-19 patients according to mechanical ventilation and extracorporeal membrane oxygenation use: The LIFE study
Source: PLoS One. 2026 Mar 26;21(3):e0345939. doi: 10.1371/journal.pone.0345939 (PMC13020807; doi:10.1371/journal.pone.0345939)
Supplement: S1 File — Cox Regression Analysis of 180-Day Post-Discharge Mortality with Age Groups. Concordance = 0.718 (standard error = 0.011). CI, confidence interval; ECMO, extracorporeal membrane oxygenation; LOS, length of stay; MV, mechanical ventilation. S2 Table. Generalized Linear Model Analysis of 180-Day Post-Discharge Total Medical Expenditures with Age Groups. CI, confidence interval; ECMO, extracorporeal membrane oxygenation; LOS, length of stay; MV, mechanical ventilation. S3 Table. Cox Regression Analysis of 180-Day Post-Discharge Mortality with Charlson Comorbidity Index Scores. Concordance = 0.70 (standard error = 0.012). CI, confidence interval; ECMO, extracorporeal membrane oxygenation; LOS, length of stay; MV, mechanical ventilation. S4 Table. Cox Regression Analysis of 180-Day Post-Discharge Mortality with Elixhauser Comorbidity Index Scores. Concordance = 0.70 (standard error = 0.012). CI, confidence interval; ECMO, extracorporeal membrane oxygenation; LOS, length of stay; MV, mechanical ventilation. S5 Table. Generalized Linear Model Analysis of 180-Day Post-Discharge Total Medical Expenditures with Charlson Comorbidity Index Scores. CI, confidence interval; ECMO, extracorporeal membrane oxygenation; LOS, length of stay; MV, mechanical ventilation. S6 Table. Generalized Linear Model Analysis of 180-Day Post-Discharge Total Medical Expenditures with Elixhauser Comorbidity Index Scores. CI, confidence interval; ECMO, extracorporeal membrane oxygenation; LOS, length of stay; MV, mechanical ventilation. S7 Table. Cox Regression Analysis of 180-Day Post-Discharge Mortality with COVID-19 Variant Periods. Concordance = 0.718 (standard error = 0.011). CI, confidence interval; ECMO, extracorporeal membrane oxygenation; LOS, length of stay; MV, mechanical ventilation. S8 Table. Generalized Linear Model Analysis of 180-Day Post-Discharge Total Medical Expenditures with COVID-19 Variant Periods. CI, confidence interval; ECMO, extracorporeal membrane oxygenation; LOS, length o [file pone.0345939.s001.zip › Supporting Information/S1 Table.docx]

**S1 Table. Cox Regression Analysis of 180-Day Post-Discharge Mortality with Age Groups**.

| **Independent Variables** | **Hazard Ratio** | **95% CI** | ***p*-value** |
| --- | --- | --- | --- |
| MV/ECMO (ref: Non-MV/ECMO) | 1.52 | 1.17–1.98 | 0.002 |
| Age, 65–74 years (ref: <65 years)  Age, ≥75 years (ref: <65 years) | 1.83  3.21 | 1.15–2.90  2.10–4.90 | 0.01  <0.001 |
| Male (ref: female) | 1.09 | 0.91–1.32 | 0.35 |
| Obesity | 0.69 | 0.22–2.18 | 0.53 |
| LOS | 1.01 | 1.01–1.02 | <0.001 |
| Hospitalization expenditure | 1.00 | 1.00–1.00 | 0.004 |
| Delirium on admission | 1.04 | 0.78–1.40 | 0.79 |
| Hypertension | 0.80 | 0.66–0.98 | 0.032 |
| Diabetes | 1.10 | 0.89–1.37 | 0.38 |
| Lower respiratory disease | 1.45 | 1.08–1.95 | 0.014 |
| Heart disease | 1.09 | 0.90–1.33 | 0.38 |
| Kidney disease | 1.42 | 0.98–2.06 | 0.06 |
| Cerebrovascular disease | 0.98 | 0.78–1.23 | 0.89 |
| Dementia | 1.82 | 1.36–2.44 | <0.001 |
| Cancer | 1.89 | 1.54–2.32 | <0.001 |
| Liver disease | 1.78 | 1.09–2.87 | 0.02 |
| Concordance = 0.718 (standard error = 0.011). CI, confidence interval; ECMO, extracorporeal membrane oxygenation; LOS, length of stay; MV, mechanical ventilation. | | | |
