# Supplementary material for: Comparison of post-discharge mortality and medical expenditures in COVID-19 patients according to mechanical ventilation and extracorporeal membrane oxygenation use: The LIFE study
Source: PLoS One. 2026 Mar 26;21(3):e0345939. doi: 10.1371/journal.pone.0345939 (PMC13020807; doi:10.1371/journal.pone.0345939)
Supplement: Attachment — Submitted filename: Review .pdf [file pone.0345939.s003.pdf]

The introduction effectively highlights the long-term health and economic burdens experienced by survivors of severe COVID-19, emphasizing the public health relevance of the study. The authors clearly outline their hypothesis and objectives, focusing on the effects of mechanical ventilation (MV) and extracorporeal membrane oxygenation (ECMO) on 180-day post-discharge outcomes. While ECMO is included in the hypothesis and objectives, prior studies specifically examining ECMO use and long-term outcomes are not discussed.
Suggestion: Incorporating background literature on ECMO would strengthen the rationale for the study.

The Methods section is transparent and well-aligned with the study objectives. Inclusion criteria, follow-up period, and case identification using ICD-10 and Japanese medical fee codes are described in sufficient detail to allow reproducibility. The analytical approach is appropriate, and the extensive sensitivity analyses enhance the credibility of the results. Since MV and ECMO represent different levels of disease severity and resource utilization,
Suggestion: Performing a separate analysis for MV-only and ECMO patients could improve the methodological rigor.

In the Results section, the secondary and sensitivity analyses demonstrate the associations of MV/ECMO use with mortality and expenditures are robust across multiple analytical approaches, including consideration of survivorship bias, age stratification, comorbidity indices.

The discussion is thorough, well-referenced, and demonstrates clear scientific reasoning. Some sentences are lengthy and complex, which may affect readability. Breaking up long paragraphs, especially when moving between topics such as mortality, comorbidities, and costs, would make the discussion easier to follow.
