# Supplementary material for: Comparison of post-discharge mortality and medical expenditures in COVID-19 patients according to mechanical ventilation and extracorporeal membrane oxygenation use: The LIFE study
Source: PLoS One. 2026 Mar 26;21(3):e0345939. doi: 10.1371/journal.pone.0345939 (PMC13020807; doi:10.1371/journal.pone.0345939)
Supplement: Attachment — Submitted filename: Response to Reviewers.docx [file pone.0345939.s004.docx]

**Response to the Editor and Reviewer**

August 14, 2025

**To Dr. Campbell and the editorial team at *PLOS ONE*,**

We would like to express our gratitude to you and the reviewer for the valuable input. We have made extensive revisions throughout the manuscript in accordance with the comments and advice, and would like to re-submit our revised manuscript for further consideration. Our point-by-point responses and revisions to the editor’s and reviewer’s comments are detailed below. In addition to the specific revisions, the entire manuscript has been professionally re-edited for language and readability. Revisions to the manuscript are indicated with red text. Page and line numbers refer to those in the track changes version with “All Markup” displayed.

The title has been changed to “**Comparison of post-discharge mortality and medical expenditures in COVID-19 patients according to mechanical ventilation and extracorporeal membrane oxygenation use: The LIFE Study**”. While our study exposure included both MV and ECMO use, the original title only included MV. We believe the revised title is a better indication of our research.

***Journal Requirements:***

***Comment #1***

*1. Please ensure that your manuscript meets PLOS ONE's style requirements, including those for file naming.*

**Response to Comment #1:**

As instructed, we have formatted our manuscript, figures, tables, and supporting information files to ensure that they fully comply with the journal’s requirements.

***Comment #2***

*2. PLOS requires an ORCID iD for the corresponding author in Editorial Manager on papers submitted after December 6th, 2016. Please ensure that you have an ORCID iD and that it is validated in Editorial Manager.*

**Response to Comment #2:**

The corresponding author has confirmed that his ORCID iD is registered and successfully validated in the Editorial Manager system.

***Comment #3***

*3. For studies involving third-party data, we encourage authors to share any data specific to their analyses that they can legally distribute. PLOS recognizes, however, that authors may be using third-party data they do not have the rights to share. When third-party data cannot be publicly shared, authors must provide all information necessary for interested researchers to apply to gain access to the data.*

**Response to Comment #3:**

Thank you for pointing this out. We include the following information in the Data Availability Statement upon submission:

**Data Availability Statement**

The data used in this study were acquired under agreements between Kyushu University and the participating municipalities, which stipulate that the data can only be used by authorized research institutions and cannot be shared with third parties. However, research institutions that have entered into agreements with the authorized research group in Kyushu University may access the data. Please contact the Joint Research Department of Kyushu University ([ijkkyoudou@jimu.kyushu-u.ac.jp](mailto:ijkkyoudou@jimu.kyushu-u.ac.jp)) for inquiries regarding data access.

***Comment #4***

*4. Please include your full ethics statement in the ‘Methods’ section of your manuscript file. In your statement, please include the full name of the IRB or ethics committee who approved or waived your study, as well as whether or not you obtained informed written or verbal consent. If consent was waived for your study, please include this information in your statement as well.*

**Response to Comment #4:**

As instructed, we have included the full ethics statement in the Methods section as follows (Page 16, Lines 345–350 in the track changes version of the manuscript):

**Ethics statement**

This study was conducted in accordance with the principles of the Declaration of Helsinki. The study was approved by the Kyushu University Institutional Review Board for Clinical Research (Approval no. 22114-03). The review board waived the requirement for informed consent due to the study’s retrospective nature and because all records were de-identified and fully anonymized before being received by the authors.

***Comment #5***

*5. Please upload a copy of supplementary material which you refer to in your text on page 7.*

**Response to Comment #5**

The erroneous reference to supplementary material has been deleted from the Statistical analysis subsection. We apologize for the error.

***Reviewer #1***

-------------------------------------------------------------------------------------------------------------------

***Overall recommendation:***

***Comment #1***

*-While the conclusions are supported by the data, the manuscript should emphasize that associations do not prove causation due to observational design.
-Consider discussing potential residual confounding and unmeasured severity indicators.
-Future studies with clinical data and multi-center cohorts would strengthen evidence.*

**Response to Comment #1:**

To Dr. Goswami: We are sincerely grateful for your time and effort in reviewing our work. Your insightful and constructive comments have greatly helped us improve the manuscript’s clarity and rigor. We have individually addressed each point below. Revisions to the manuscript are indicated with red text. Page and line numbers refer to those in the track changes version with “All Markup” displayed.

In addition to the specific revisions, the entire manuscript has been professionally re-edited for language and readability. The title has been changed to “**Comparison of post-discharge mortality and medical expenditures in COVID-19 patients according to mechanical ventilation and extracorporeal membrane oxygenation use: The LIFE Study**”. While our study exposure included both MV and ECMO use, the original title only included MV. We believe the revised title is a better indication of our research.

**Comment 1.1:**

*-While the conclusions are supported by the data, the manuscript should emphasize that associations do not prove causation due to observational design.*

**Response 1.1:**

We agree on this critical point. As instructed, we acknowledge this as the first limitation in the Discussion (Page 34, Lines 638–641 in the track changes version of the manuscript)：

First, this was an observational study conducted in a single Japanese municipality. Therefore, the analyses do not prove causation between severe COVID-19 and the study outcomes, and our findings may have limited generalizability.

**Comment 1.2:**

*-Consider discussing potential residual confounding and unmeasured severity indicators.*

**Response 1.2:**

Thank you for the important suggestion. We acknowledge that residual confounding from unmeasured variables, such as clinical severity indicators, is a key limitation of our study. We have revised the Limitations section to address this point (Pages 34–35, Lines 641–647 in the track changes version of the manuscript):

Second, COVID-19 severity was inferred solely from claims data, potentially leading to misclassifications due to coding errors or incomplete claims. These misclassifications may also have affected the identification of comorbidities. In addition, the claims data did not include detailed clinical information, such as laboratory data (e.g., C-reactive protein) and severity markers (e.g., oxygen saturation and APACHE II scores). The absence of these variables may have led to residual confounding, potentially distorting our results.

**Comment 1.3:**

*-Future studies with clinical data and multi-center cohorts would strengthen evidence.*

**Response 1.3:**

We completely agree with your perspective on future research directions. We have incorporated this suggestion into the conclusion of our Limitations section to highlight the need for more robust future studies (Page 35, Lines 652–656 in the track changes version of the manuscript):

To build upon our findings, future research should aim to address these limitations. Specifically, prospective multi-center cohort studies incorporating detailed clinical data and patient-reported outcomes would provide a more comprehensive understanding of the long-term trajectory of severe COVID-19 survivors.

-------------------------------------------------------------------------------------------------------------------

**Abstract:**

***Comment #2***

*-The phrase "Outcomes of health condition and economic of patients" is awkward and unclear. Suggestion: Outcomes related to health status and economic burden among patients who experienced critical COVID-19 remain insufficiently studied.
-Use consistent terminology (e.g., "mechanical ventilation" vs. "ventilator use").
-Avoid redundancy (e.g., "COVID-19 patients who did or did not require mechanical ventilation in ICU or ED" can be simplified).*

**Response to Comment #2:**

We are grateful for these insightful suggestions to improve the abstract’s clarity and conciseness. The abstract has been extensively revised based on your suggestions.

**Comment 2.1:**

*- The phrase "Outcomes of health condition and economic of patients" is awkward and unclear. Suggestion: Outcomes related to health status and economic burden among patients who experienced critical COVID-19 remain insufficiently studied.*

**Response 2.1:**

We have adopted your suggestion as the opening sentence of the abstract to better articulate the study’s background regarding health and economic outcomes (Page 3, Lines 31–32 in the track changes version of the manuscript):

Outcomes related to health status and economic burden among patients who experienced critical COVID-19 remain insufficiently studied.

**Comment 2.2:**

*- Use consistent terminology (e.g., "mechanical ventilation" vs. "ventilator use").*

**Response 2.2:**

As instructed, we have standardized the terminology to “mechanical ventilation”, and use the abbreviation “MV” throughout the manuscript to ensure consistency. In addition, our two study groups are now designated “MV/ECMO” and “non-MV/ECMO” to reflect that the exposure included the use of MV *or* ECMO.

**Comment 2.3:**

*- Avoid redundancy (e.g., "COVID-19 patients who did or did not require mechanical ventilation in ICU or ED" can be simplified).*

**Response 2.3:**

The abstract has been extensively revised, and that phrase has been rewritten as follows (Page 3, Lines 35–37 in the track changes version of the manuscript):

…COVID-19 patients according to their use of mechanical ventilation (MV) or extracorporeal membrane oxygenation (ECMO) during hospitalization.

-------------------------------------------------------------------------------------------------------------------

**Introduction:**

***Comment #3***

*Some sentences are verbose or repetitive. Use consistent terminology (e.g., "severe COVID-19" rather than "severe cases of COVID-19"). Clarify ambiguous phrases (e.g., "patients with severe COVID-19 who are discharged from an ICU may continue to have physical, mental, and cognitive symptoms" — specify "post-discharge").*

*Example for first paragraph:*

*The COVID-19 pandemic has led to a surge of critically ill patients requiring intensive care worldwide [1]. Patients discharged from ICUs after severe COVID-19 often experience persistent physical, mental, and cognitive impairments [2,3], and face increased risks of poor prognosis and higher healthcare costs post-discharge [4,5]*

*ventilation in ICU or ED" can be simplified).*

**Response to Comment #3:**

We are very grateful for the meticulous feedback and for taking the time to provide an example of how to improve the writing. We agree that our original phrasing was at times repetitive and unclear. In response, we have:

1. Standardized the terminology to "severe COVID-19"

2. Clarified ambiguous phrases

For example, we have adopted your suggested revision for the first paragraph, which has significantly improved its clarity (Page 5, Lines 79–82 in the track changes version of the manuscript):

The COVID-19 pandemic led to a surge in critically ill patients requiring intensive care worldwide [1]. Patients discharged from intensive care units (ICUs) after severe COVID-19 often experience persistent physical, mental, and cognitive impairments [2,3], and face increased risks of poor prognosis and higher healthcare costs post-discharge [4,5].

The rest of the Introduction has also been rewritten to improve its flow, consistency, and readability.

**-------------------------------------------------------------------------------------------------------------------**

**Methods:**

***Comment #4***

***Methods:***

*Clarify some complex sentences for readability.*

*Use consistent terminology for insurance systems.*

*Avoid passive voice where possible.*

*Clarify exclusion criteria and study population flow. Correct minor grammar issues (e.g., "We have accessed between..." → "Data were accessed between...").*

***Example revision snippet:*** *This cohort study used data from the LIFE Study, a research database managed by Kyushu University, containing claims data from Japan's National Health Insurance System (ages 0–74) and the Latter-Stage Elderly Healthcare System (ages ≥75 and 65–74 with certain diseases) for residents of a single municipality [11]. Data were accessed between August 1, 2023, and February 1, 2024. Patient identifiers were anonymized to protect privacy.*

**Response to Comment #4:**

Thank you for the thorough and helpful comments. We have carefully revised the entire Methods section to improve its clarity, consistency, and readability by incorporating your feedback, such as the paragraph below (Pages 8–9, Lines 160–167 in the track changes version of the manuscript):

This retrospective cohort study used data from the Longevity Improvement & Fair Evidence (LIFE) Study, a research database managed by Kyushu University [11]. The study data contained medical claims data from Japan’s National Health Insurance System (ages 0–74), Latter-Stage Older Persons Healthcare System (ages ≥75 and 65–74 with certain diseases), and the Public Assistance System for residents of a single municipality. In addition, we received Basic Resident Register data for this municipality. Data were accessed between August 1, 2023 and February 1, 2024. Patient identifiers were anonymized to protect privacy.

We have also clarified the exclusion criteria as follows (Pages 10–11, Lines 202–215 in the track changes version of the manuscript):

We first excluded individuals who fulfilled any of the following criteria: (i) previous hospitalization within 6 months before the index hospitalization, (ii) duplicate patient ID records in the study data, and (iii) age <18 years. To analyze the primary outcome of mortality, we also excluded (iv) patients who died during hospitalization or on the discharge date and (v) patients with a length of stay (LOS) of only 1 day. To analyze the secondary outcome of total medical expenditures, we further excluded (vi) patients who died within 180 days post-discharge to specifically assess the ongoing costs for survivors and avoid the distinct expenditure patterns associated with end-of-life care [12] and (vii) patients with no medical claims data within 180 days post-discharge. As our study cohort was limited to persons who resided within the study municipality throughout the follow-up period, an absence of claims data would signify that these individuals did not use any insured medical services. The exclusion of these zero-expenditure patients allowed for a more accurate analysis of the distribution and determinants of expenditures by focusing on the population that used healthcare services post-discharge.

***Comment #5***

*-Severity of COVID-19 is inferred indirectly via mechanical ventilation/ECMO use and ICU billing codes, which may not fully capture clinical severity or differences in care.
Suggestion: Consider incorporating additional clinical severity indicators if available (e.g., oxygen saturation, laboratory markers, APACHE II scores).
If unavailable, explicitly acknowledge this limitation and discuss potential residual confounding.
if possible - Use propensity score methods or inverse probability weighting to better balance groups on observed covariates and reduce confounding bias.*

**Response to Comment #5:**

Thank you for raising this critical methodological point concerning the measurement of disease severity and the potential for residual confounding. We agree that this is an inherent limitation of our study. As you surmised, our claims database does not contain detailed clinical indicators, and we have therefore acknowledged this limitation in the manuscript (Pages 34–35, Lines 641–647 in the track changes version of the manuscript).

Second, COVID-19 severity was inferred solely from claims data, potentially leading to misclassifications due to coding errors or incomplete claims. These misclassifications may also have affected the identification of comorbidities. In addition, the claims data did not include detailed clinical information, such as laboratory data (e.g., C-reactive protein) and severity markers (e.g., oxygen saturation and APACHE II scores). The absence of these variables may have led to residual confounding, potentially distorting our results.

We also appreciate the suggestion to use propensity score methods. We carefully considered this approach during the study design phase. However, given that the primary source of potential confounding—detailed clinical severity—is based on unobserved variables in our claims data, we determined that propensity scores would not be suitable for our study.

***Comment #6***

*-Address Potential Selection Bias - Excluding patients who died during hospitalization or within 180 days post-discharge for expenditure analysis may introduce survivorship bias.
Suggestion:
Discuss this potential bias explicitly.
Consider sensitivity analyses including these patients or using methods to account for censored data (e.g., competing risks models).
Justify the exclusion of patients without claims data within 180 days (e.g., due to relocation or loss to follow-up).*

**Response to Comment #6:**

Thank you for raising these important methodological issues. We agree that investigating the potential for survivorship bias is essential for validating our conclusions. Based on your suggestion, we have taken the following steps and conducted a new sensitivity analysis to test the robustness of our expenditure findings.

**Comment 6.1:**

*-Address Potential Selection Bias - Excluding patients who died during hospitalization or within 180 days post-discharge for expenditure analysis may introduce survivorship bias.*

**Response 6.1:**

As you rightly pointed out, our inability to treat mortality as a competing risk is an important limitation of this study. We have added this as a limitation (Page 35, Lines 649–652 in the track changes version of the manuscript):

Finally, our analysis of expenditures excluded patients who died within 180 days post-discharge, which may have introduced survivorship bias. Although a sensitivity analysis confirmed the robustness of our results, we could not account for death as a competing risk for cost estimation.

**Comment 6.2:**

*Consider sensitivity analyses including these patients or using methods to account for censored data (e.g., competing risks models).*

**Response 6.2:**

**Sensitivity Analysis:**

Thank you for the suggestion. We have added a sensitivity analysis in which we compared medical expenditures between the MV/ECMO and non-MV/ECMO groups while including the 461 patients who died within 180 days after discharge. (As the study outcome was post-discharge expenditures, we did not include patients who died during hospitalization.) We have added the following to the Methods (Page 15 Lines 323–328 in the track changes version of the manuscript):

**Sensitivity analysis**

First, we addressed the possibility of survivorship bias by conducting a sensitivity analysis of total medical expenditures in a cohort that also included the 461 patients who died within 180 days post-discharge. In this cohort, we compared the median total medical expenditures between the MV/ECMO and non-MV/ECMO groups using the Mann–Whitney *U* test.

**Comment 6.3:**

*Justify the exclusion of patients without claims data within 180 days (e.g., due to relocation or loss to follow-up).*

**Response 6.3:**

As instructed, we have clarified the rationale for handling patients without claims data. Our study design, which tracked only non-relocating residents, allowed for a more accurate analysis of the distribution and determinants of medical expenditures by focusing on the population that used healthcare services post-discharge (Page 9, Lines 181–186 in the track changes version of the manuscript):

From the Basic Resident Register data, we first identified residents who did not relocate from the study municipality between October 1, 2019, and March 31, 2022. This was because each municipal government serves as the insurer for residents enrolled in the target insurance systems, and individuals who move to a different municipality would be lost to follow-up.

(Pages 10–11, Lines 210–215 in the track changes version of the manuscript):

As our study cohort was limited to persons who resided within the study municipality throughout the follow-up period, an absence of claims data would signify that these individuals did not use any insured medical services. The exclusion of these zero-expenditure patients allowed for a more accurate analysis of the distribution and determinants of expenditures by focusing on the population that used healthcare services post-discharge.

***Comment #7***

*Expand on Comorbidity Assessment*

*- Comorbidities are identified using ICD-10 codes up to 180 days prior to hospitalization, but the method for defining presence (e.g., single code vs. multiple codes) is not detailed.
Suggestion: Specify the algorithm or criteria used to define comorbidities (e.g., at least one inpatient or two outpatient claims).
Consider using validated comorbidity indices (e.g., Charlson or Elixhauser) as composite measures. Discuss potential misclassification due to coding errors or incomplete claims.*

**Response to Comment #7:**

As suggested, we have revised the manuscript to expand on comorbidity assessment.

**Comment 7.1:**

*- Comorbidities are identified using ICD-10 codes up to 180 days prior to hospitalization, but the method for defining presence (e.g., single code vs. multiple codes) is not detailed.
Suggestion: Specify the algorithm or criteria used to define comorbidities (e.g., at least one inpatient or two outpatient claims).*

**Response 7.1:**

We defined a comorbidity as present if at least one corresponding ICD-10 code appeared in a claims record within the 180-day pre-hospitalization period, and have described this algorithm in the Methods section (Page 13, Lines 270–272 in the track changes version of the manuscript):

The presence of a comorbidity was defined by at least one relevant ICD-10 code appearing in an inpatient or outpatient claims record during this look-back period.

**Comment 7.2:**

*- Consider using validated comorbidity indices (e.g., Charlson or Elixhauser) as composite measures.*

**Response 7.2:**

Thank you for the valuable suggestion. We have now conducted two new sensitivity analyses using the Charlson and Elixhauser comorbidity scores in place of individual conditions in our multivariable models (Page 16, Lines 333–336 in the track changes version of the manuscript):

Third, to assess the impact of comorbidity burden, Charlson Comorbidity Index and Elixhauser Comorbidity Index scores (i.e., composite measures) were calculated for each patient and included as covariates instead of individual conditions in the analytical models.

**Comment #7.3:**

*Discuss potential misclassification due to coding errors or incomplete claims.*

**Response 7.3:**

We have expanded the Limitations section to more explicitly discuss the potential for misclassification when using claims data (Pages 34–35, Lines 641–647 in the track changes version of the manuscript):

Second, COVID-19 severity was inferred solely from claims data, potentially leading to misclassifications due to coding errors or incomplete claims. These misclassifications may also have affected the identification of comorbidities. In addition, the claims data did not include detailed clinical information, such as laboratory data (e.g., C-reactive protein) and severity markers (e.g., oxygen saturation and APACHE II scores). The absence of these variables may have led to residual confounding, potentially distorting our results.

***Comment #8***

*Clarify Definition and Timing of Medical Expenditures - Medical expenditures are defined from the month following discharge through 180 days, excluding the discharge month.
Suggestion: Provide rationale for excluding the discharge month expenditures (e.g., to separate inpatient from outpatient costs).
Clarify whether expenditures include all healthcare costs (inpatient, outpatient, pharmacy) or only specific categories.
Consider analyzing expenditures by category to identify drivers of cost differences.*

**Response to Comment #8:**

Thank you for these helpful suggestions to clarify our expenditure analysis. We have revised the manuscript accordingly.

**Comment 8.1:**

*Clarify Definition and Timing of Medical Expenditures - Medical expenditures are defined from the month following discharge through 180 days, excluding the discharge month.
Suggestion: Provide rationale for excluding the discharge month expenditures (e.g., to separate inpatient from outpatient costs).*

**Response 8.1:**

Thank you for the suggestion. We have clarified in the Methods section that the discharge month was excluded because Japanese claims data for each month bundles hospitalization expenditures, making it impossible to isolate post-discharge expenditures from the index hospitalization (Page 12, Lines 238–246 in the track changes version of the manuscript):

For expenditures, the 180-day period began from the month following discharge (excluding the discharge month). Under the Japanese healthcare system, medical claims data are collected monthly, and the hospitalization expenditures for each month are bundled together. To distinguish between the expenditures for the index hospitalization and new expenditures incurred independently in the post-discharge period, we did not include expenditures in the discharge month.

**Comment 8.2:**

*Clarify whether expenditures include all healthcare costs (inpatient, outpatient, pharmacy) or only specific categories.*

**Response 8.2:**

As instructed, we have specified in the Methods section that the analyzed expenditures include all insurance-covered inpatient, outpatient, pharmacy, and dental costs (Pages 11–12, Lines 232–234 in the track changes version of the manuscript):

The primary outcome was 180-day post-discharge mortality, and the secondary outcome was 180-day post-discharge total medical expenditures (encompassing all insurance-covered inpatient, outpatient, pharmacy, and dental costs).

**Comment 8.3:**

*Consider analyzing expenditures by category to identify drivers of cost differences.*

**Response 8.3:**

Thank you for this constructive suggestion. We believe that this additional analysis substantially strengthens our manuscript by providing a more granular view of the post-discharge economic burden, and are grateful for your suggestion. We have added the following to the Methods and Results (Page 15, Lines 315–318 & Page 27, Lines 468–475 in the track changes version of the manuscript):

In addition, the number of hospital readmissions and the components of total medical expenditures (inpatient, outpatient, pharmacy, and dental) were evaluated as secondary analyses. The median number of hospital readmissions and costs for each component were compared between the groups using the Mann–Whitney *U* test.

**Secondary analyses: readmission and cost components**

The median number of hospital readmissions within 180 days post-discharge was significantly higher in the MV/ECMO group than in the non-MV/ECMO group (1.0 [IQR: 0.0, 1.0] vs. 0.0 [IQR: 0.0, 1.0], *p*<0.001). Next, the analysis of cost components showed that median inpatient costs were significantly higher in the MV/ECMO group than in the non-MV/ECMO group ($671 vs. $359, *p*<0.001). In contrast, there were no significant intergroup differences in outpatient costs ($69 vs. $67, *p*=0.607), pharmacy costs ($45 vs. $40, *p*=0.393), or dental costs ($0 vs. $0, *p*=0.313).

***Comment #9***

*If time allows and possible - Consider Additional Outcomes or Subgroup Analyses
Suggestion:
Include other relevant outcomes such as readmission rates, quality of life, or functional status if data permit.
Perform subgroup analyses by age groups, comorbidity burden, or variant periods (alpha vs. delta) to explore heterogeneity.
Explore time-varying covariates or longitudinal expenditure patterns.*

**Response to Comment #9:**

Thank you for the constructive suggestions.

**Comment 9.1:**

*Include other relevant outcomes such as readmission rates, quality of life, or functional status if data permit.*

**Response 9.1:**

As recommended, we have conducted an additional analysis on the number of hospital readmissions within 180 days, which was feasible using our dataset (n=3,407). We have added this new analysis (along with a cost component analysis) to the Methods section, and added its findings to the Results section (Page 15, Lines 315–318 & Page 27, Lines 468–475 in the track changes version of the manuscript):

In addition, the number of hospital readmissions and the components of total medical expenditures (inpatient, outpatient, pharmacy, and dental) were evaluated as secondary analyses. The median number of hospital readmissions and costs for each component were compared between the groups using the Mann–Whitney *U* test.

**Secondary analyses: readmission and cost components**

The median number of hospital readmissions within 180 days post-discharge was significantly higher in the MV/ECMO group than in the non-MV/ECMO group (1.0 [IQR: 0.0, 1.0] vs. 0.0 [IQR: 0.0, 1.0], *p*<0.001). Next, the analysis of cost components showed that median inpatient costs were significantly higher in the MV/ECMO group than in the non-MV/ECMO group ($671 vs. $359, *p*<0.001). In contrast, there were no significant intergroup differences in outpatient costs ($69 vs. $67, *p*=0.607), pharmacy costs ($45 vs. $40, *p*=0.393), or dental costs ($0 vs. $0, *p*=0.313).

**Comment 9.2:**

*Perform subgroup analyses by age groups, comorbidity burden, or variant periods (alpha vs. delta) to explore heterogeneity.*

**Response 9.2:**

Thank you for the suggestions. We agree that subgroup analyses by age groups, comorbidity burden, and variant periods could provide important insight into the possible heterogeneity in our results.

However, our study cohort may not be suitable for these subgroup analyses. First, the cohort was skewed toward older adults (which aligns with the general patient profile admitted to ICUs or EDs during the pandemic in Japan), and grouping patients according to age would result in disproportionately small sample sizes for younger patients. This potential imbalance in groups would also affect a subgroup analysis of COVID-19 variant periods: our cohort comprised patients hospitalized between April 1, 2020 and September 30, 2021. Given that the Delta variant period began from July 1, 2021 in Japan, this subgroup would only have 3 months of data (compared to the 15 months of the pre-Delta variant period). Conducting subgroup analyses with unbalanced groups runs the risk of introducing bias into the results. For comorbidity burden, it would be difficult to determine appropriate cut-offs to delineate the subgroups.

Nevertheless, we feel that our study should examine your suggested parameters. Instead of subgroup analyses, we have examined these three parameters as sensitivity analyses. We apologize that we are unable to perform the subgroup analyses as recommended, and hope that these additions are acceptable.

**1. Age groups:**

We stratified our cohort into three age groups (<65 years, 65–74 years, and ≥75 years), and re-performed the primary analyses for mortality and expenditures with these age groups instead of age as a continuous variable. The results are presented as new supplementary tables (S1 and S2 Tables).

**2. Comorbidity burden:**

To assess the impact of comorbidity burden, we have now incorporated the Charlson Comorbidity Index and Elixhauser Comorbidity Index scores as covariates (in place of individual conditions) in our primary Cox regression and generalized linear models. The results are presented as new supplementary tables (S3 to S6 Tables)

**3. Variant period:**

To account for the potential influence of the predominant viral variant, we used a variable to distinguish between the pre-Delta and Delta-dominant periods in Japan. This variable was included as a covariate in our analytical models, and the results shown as new supplementary tables (S7 and S8 Tables).

　The detailed methods for these additional analyses have been added to the revised manuscript (Page 16, Lines 329–343 in the track changes version of the manuscript):

Second, we stratified our cohort into three age groups (<65 years, 65–74 years, and ≥75 years), and re-performed the primary analyses for mortality and expenditures with these age groups instead of age as a continuous variable. The age groups were set based on a previous study of older adults with COVID-19 in Japan [19].

Third, to assess the impact of comorbidity burden, Charlson Comorbidity Index and Elixhauser Comorbidity Index scores (i.e., composite measures) were calculated for each patient and included as covariates instead of individual conditions in the analytical models.

Fourth, to assess the potential effects of different COVID-19 variant periods, the study cohort was stratified into two groups according to patient admission date: the pre-Delta variant period (April 1, 2020, to June 30, 2021) and the Delta variant period (July 1, 2021, to September 30, 2021). These periods were based on the documented pandemic timeline in Japan [20]. To assess whether the impact of MV/ECMO use on the outcomes was modified by the variant period, an interaction term between the Delta variant period and MV/ECMO was included in the analytical models.

- The following references have been added (Page 38, Lines 730–736):

19. Goswami H, Tajima A, Matsuki T, Puenpatom A. Cost-effectiveness analysis of molnupiravir versus best supportive care for the treatment of outpatient COVID-19 in high-risk older adults in Japan. Pharmacoeconom Open. 2025; 9(4): 571–84. doi.org/10.1007/s41669-025-00578-y

20. Ogata T, Tanaka H, Irie F, Hirayama A, Takahashi Y. Shorter incubation period among unvaccinated Delta variant Coronavirus disease 2019 patients in Japan. Int J Environ Res Public Health. 2022; 19(3): 1127. doi.org/10.3390/ijerph19031127

**Comment #9.3:**

*Explore time-varying covariates or longitudinal expenditure patterns.*

**Response to Comment #9.3:**

Thank you for the suggestion. We agree that exploring time-varying covariates and longitudinal expenditure patterns would provide a more dynamic and deeper understanding of the topic. However, due to the limitations of our dataset, we are unable to implement these analyses. The data available to us do not allow for the accurate tracking of covariates over time or for a detailed longitudinal analysis of expenditure patterns that this approach would require. We believe this is an important and promising direction for future research, and plan to address this specific question in a subsequent study with a more suitable longitudinal dataset.

-------------------------------------------------------------------------------------------------------------------

**Results:**

***Comment #10***

*-Address Missing Data and Sensitivity Analyses
The results do not mention handling of missing data or sensitivity analyses.
Suggestion: State whether missing data were present and how they were handled.
Consider reporting sensitivity analyses if performed (e.g., including patients who died within 180 days for expenditures).*

**Response to Comment #10:**

Thank you for your valuable feedback regarding the handling of missing data and the reporting of sensitivity analyses. We have revised the manuscript to improve the transparency and robustness of our Methods and Results.

**Comment 10.1:**

*-Address Missing Data and Sensitivity Analyses
The results do not mention handling of missing data or sensitivity analyses.
Suggestion: State whether missing data were present and how they were handled.*

**Response 10.1:**

**Handling of Missing Data:**

We have clarified in the Methods section that there were no substantial missing data for the variables used in our analyses. The only data cleaning performed was the removal of a small number of duplicate patient IDs. We have described these points in a new subsection titled "Data cleaning and handling of missing data" (Page 9 Lines 169–173 in the track changes version of the manuscript):

**Data cleaning and handling of missing data**

No substantial missing values were found in the study variables (e.g., age, sex, comorbidities, and expenditures). The only data cleaning performed was the removal of duplicate patient ID records to ensure that each individual was included only once in the analysis.

**Comment 10.2:**

*Consider reporting sensitivity analyses if performed (e.g., including patients who died within 180 days for expenditures).*

**Response 10.2:**

Based on your suggestions, we have performed several additional analyses. These include:

- Secondary analyses to examine the differences in the number of hospital readmissions and cost components between the study groups
- Sensitivity analyses including patients who died within 180 days for the expenditure analysis, age groups, comorbidity indices, and variant periods

In addition to the above, we have added the Charlson Comorbidity Index and Elixhauser Comorbidity Index scores to the baseline characteristics in Table 1. We believe this provides a more comprehensive and standardized view of the baseline comorbidity burden in both the MV/ECMO and non-MV/ECMO groups, offering a clearer picture of the patient populations being compared.

The results of these additional analyses have been added as follows:

1. Page 27, Lines 468–475 in the track changes version of the manuscript:

**Secondary analyses: readmission and cost components**

The median number of hospital readmissions within 180 days post-discharge was significantly higher in the MV/ECMO group than in the non-MV/ECMO group (1.0 [IQR: 0.0, 1.0] vs. 0.0 [IQR: 0.0, 1.0], *p*<0.001). Next, the analysis of cost components showed that median inpatient costs were significantly higher in the MV/ECMO group than in the non-MV/ECMO group ($671 vs. $359, *p*<0.001). In contrast, there were no significant intergroup differences in outpatient costs ($69 vs. $67, *p*=0.607), pharmacy costs ($45 vs. $40, *p*=0.393), or dental costs ($0 vs. $0, *p*=0.313).

2. Pages 27–29, Lines 477–512 in the track changes version of the manuscript:

**Sensitivity analyses**

First, we compared the median total medical expenditures between the MV/ECMO and non-MV/ECMO groups in a cohort that included patients who died within 180 days post-discharge (n=3,868). The median expenditures of the MV/ECMO group were 2.2 times higher than that of the non-MV/ECMO group ($8,285 [IQR: $2,054–$23,628] vs. $3,792 [IQR: $1,256–$14,160], *p*<0.001).

Second, a Cox regression analysis was performed to examine the associations of the age groups with mortality (S1 Table). After adjusting for the other covariates, older age was found to be a strong predictor of mortality. Compared to patients aged <65 years, the hazard for mortality was significantly higher for patients aged 65–74 years (HR: 1.83, 95% CI: 1.15–2.90), and was even more pronounced for those aged ≥75 years (3.21, 2.10–4.90). Similarly, a generalized linear model was used to examine the associations of the age groups with total medical expenditures (S2 Table). Compared to patients aged <65 years, expenditures were significantly higher for those aged 65–74 years (Exp[β]: 1.37, 95% CI: 1.18–1.58) and ≥75 years (1.53, 1.34–1.74). In both analyses, MV/ECMO use remained significantly associated with the outcomes.

Third, we assessed the alternate impact of comorbidities on the outcomes by using the Charlson Comorbidity Index (S3–S4 Tables) and the Elixhauser Comorbidity Index (S5–S6 Tables) in place of individual conditions in the analytical models. In the Cox regression models, both the Charlson score (HR: 1.10, 95% CI: 1.03–1.17) and Elixhauser score (1.07, 1.02–1.12) were positively associated with mortality. Similarly, the generalized linear models showed that both the Charlson score (Exp[β]: 1.12, 95% CI: 1.08–1.16) and Elixhauser score (1.07, 1.05–1.10) were positively associated with total medical expenditures. MV/ECMO use remained significantly associated with the outcomes for both comorbidity indices.

Fourth, we assessed the potential influence of the COVID-19 Delta and pre-Delta variant periods. Neither the Delta variant period itself (*p*=0.38) nor the interaction term between the Delta variant period and MV/ECMO (*p*=0.85) were significantly associated with mortality (S7 Table), suggesting that the association between MV/ECMO and mortality did not significantly differ between the variant periods. In contrast, the interaction term between the Delta variant period and MV/ECMO was significantly associated with total medical expenditures (*p*=0.02) (S8 Table). While MV/ECMO was associated with 1.38-times higher expenditures in the pre-Delta variant period, this effect was magnified by 1.56 times during the Delta variant period. Even when accounting for COVID-19 variant periods, MV/ECMO use remained significantly associated with both outcomes.

3. Pages 41–42, Lines 786–818 in the track changes version of the manuscript (Supporting information):

**S1 Table. Cox Regression Analysis of 180-Day Post-Discharge Mortality with Age Groups.** Concordance = 0.718 (standard error = 0.011). CI, confidence interval; ECMO, extracorporeal membrane oxygenation; LOS, length of stay; MV, mechanical ventilation.

**S2 Table. Generalized Linear Model Analysis of 180-Day Post-Discharge Total Medical Expenditures with Age Groups.** CI, confidence interval; ECMO, extracorporeal membrane oxygenation; LOS, length of stay; MV, mechanical ventilation.

**S3 Table. Cox Regression Analysis of 180-Day Post-Discharge Mortality with Charlson Comorbidity Index Scores.** Concordance = 0.70 (standard error = 0.012). CI, confidence interval; ECMO, extracorporeal membrane oxygenation; LOS, length of stay; MV, mechanical ventilation.

**S4 Table. Cox Regression Analysis of 180-Day Post-Discharge Mortality with Elixhauser Comorbidity Index Scores.** Concordance = 0.70 (standard error = 0.012). CI, confidence interval; ECMO, extracorporeal membrane oxygenation; LOS, length of stay; MV, mechanical ventilation.

**S5 Table. Generalized Linear Model** **Analysis of 180-Day Post-Discharge Total Medical Expenditures with Charlson Comorbidity Index Scores.** CI, confidence interval; ECMO, extracorporeal membrane oxygenation; LOS, length of stay; MV, mechanical ventilation.

**S6 Table.** **Generalized Linear Model Analysis of** **180-Day Post-Discharge Total Medical Expenditures with Elixhauser Comorbidity Index Scores.** CI, confidence interval; ECMO, extracorporeal membrane oxygenation; LOS, length of stay; MV, mechanical ventilation.

**S7 Table.** **Cox Regression Analysis of 180-Day Post-Discharge Mortality with COVID-19 Variant Periods.** Concordance = 0.718 (standard error = 0.011). CI, confidence interval; ECMO, extracorporeal membrane oxygenation; LOS, length of stay; MV, mechanical ventilation.

**S8 Table. Generalized Linear Model Analysis of 180-Day Post-Discharge Total Medical Expenditures with COVID-19 Variant Periods.** CI, confidence interval; ECMO, extracorporeal membrane oxygenation; LOS, length of stay; MV, mechanical ventilation.

4. In addition, we have revised Fig 2 to show the cumulative incidence curves of mortality (in place of the original Kaplan–Meier survival curves) to provide a clearer and less misleading visualization. The underlying data and *p*-value remain unchanged. The Methods and Results sections have been revised to reflect this new figure.

Page 23, Lines 415–416 in the track changes version of the manuscript

**Fig 2. Cumulative Incidence Curves of 180-Day Post-Discharge Mortality.** ECMO, extracorporeal membrane oxygenation; MV, mechanical ventilation.

**New Fig 2**

**Methods section** (Page 14, Lines 294–295 in the track changes version of the manuscript)**:**

Mortality was then visualized using cumulative incidence curves, which were compared using the log-rank test.

**Results section** (Page 23, Lines 408–410 in the track changes version of the manuscript)**:**

The cumulative incidence curves of mortality were compared between the MV/ECMO and non-MV/ECMO groups (Fig 2). The MV/ECMO group had a significantly higher probability of death than the non-MV/ECMO group (log-rank *p*<0.001).

**------------------------------------------------------------------------------------------------------------------Discussion:**

***Comment #11***

*Discussion: Avoid overly long sentences. Clarify causal interpretations carefully.
Use consistent terminology. Improve flow and coherence
Example revision snippet: Our study demonstrated that COVID-19 patients requiring mechanical ventilation or ECMO had significantly higher 180-day post-discharge mortality and medical expenditures compared to those who did not require such interventions. Comorbidities including dementia, cancer, kidney disease, and cerebrovascular disease were associated with worse outcomes and increased costs. These findings align with previous studies reporting increased mortality and healthcare utilization among severe COVID-19 patients.*

**Response to Comment #11:**

Thank you for your insightful and constructive advice. We recognize that the points you raised (sentence length, causal interpretation, consistent terminology, and logical flow) are extremely important for improving the quality of our manuscript. The revision you suggested was particularly helpful for clearly conveying the main points of our discussion (we have made minor changes to reflect the additional analyses). Based on your recommendations, we have made substantial revisions to the Discussion section (Pages 29–31, Lines 526–555 in the track changes version of the manuscript):

Our study demonstrated that severe COVID-19 patients requiring MV or ECMO had significantly higher 180-day post-discharge mortality and medical expenditures compared to those who did not require such interventions. Comorbidities including dementia, cancer, kidney disease, and cerebrovascular disease were associated with poorer outcomes and increased costs. These findings were robust across multiple sensitivity analyses, and align with previous studies reporting increased mortality and healthcare utilization among severe COVID-19 patients [6-9]. Our cost component analysis revealed that the greater financial burden on patients in the MV/ECMO group was primarily driven by subsequent hospitalization costs, rather than by an increased use of outpatient, pharmacy, or dental services.

In addition to our main findings, the sensitivity analyses consistently demonstrated that older age and higher comorbidity burden are key determinants of both outcomes. The age-stratified analysis revealed that patients aged ≥65 years had significantly higher mortality and medical expenditures than younger patients, which underscores the vulnerability of this population and corroborates the findings of a previous Japanese study [19]. Furthermore, the robustness of the associations between comorbidities and the outcomes was confirmed, as both the Charlson and Elixhauser scores were significant predictors of mortality and expenditures. Our results are consistent with a large-scale study from the US that identified older age and comorbidities as primary drivers of healthcare resource utilization and costs in COVID-19 patients [21].

The interaction term between the Delta variant period and MV/ECMO was significantly associated with total medical expenditures, but not with mortality. This discrepancy may be explained by the clinical characteristics of the SARS-CoV-2 Delta variant, which was associated with more severe acute illness and a higher need for intensive care than previous variants [22]. This higher severity may also have led to a greater burden of long-term complications in survivors. A US study reported that infection during the Delta variant period was associated with a higher risk of post-acute sequelae (e.g., pulmonary and neurological disorders) than earlier variants [23]. Therefore, an increased incidence of chronic non-fatal health problems among survivors of severe Delta variant COVID-19 provides a strong potential explanation for our findings.

- The following references have been added (Pages 38–39, Lines 737–748 in the track changes version of the manuscript):

21. Liu G, Done N, Song Y, Goswami H, Wang T, Li H, et al. Healthcare resource use and costs among patients with COVID-19 in the United States. BMC Health Serv Res. 2025; 25(1): 909. doi.org/10.1186/s12913-025-12878-4

22. Twohig KA, Nyberg T, Zaidi A, Thelwall S, Sinnathamby MA, Aliabadi S, et al. Hospital admission and emergency care attendance risk for SARS-CoV-2 delta (B.1.617.2) compared with alpha (B.1.1.7) variants of concern: a cohort study. Lancet Infect Dis. 2022; 22(1): 35–42. doi.org/10.1016/S1473-3099(21)00475-8

23. Varma JK, Zang C, Carton TW, Block JP, Khullar DJ, Zhang Y, et al. Excess burden of respiratory and abdominal conditions following COVID-19 infections during the ancestral and Delta variant periods in the United States: An EHR-based cohort study from the RECOVER program. PLoS One. 2024;19(6): e0282451. doi.org/10.1371/journal.pone.0282451

**-------------------------------------------------------------------------------------------------------------------Limitation:**

***Comment #12***

*Limitations: Use formal language
Example: This study has several limitations. First, it was conducted in a single Japanese municipality, which may limit generalizability. Second, disease severity was inferred solely from claims data, potentially leading to misclassification. Third, variations in clinical management across facilities were not accounted for. Fourth, the impact of different SARS-CoV-2 variants (alpha, delta) on outcomes was not assessed. Additionally, pre-existing medical expenditures related to comorbidities prior to COVID-19 hospitalization were not evaluated.*

**Response to Comment #12:**

Thank you for providing an excellent example for the Limitations section. We agree it is a clear improvement. We have used your suggested structure as a foundation and have integrated our responses to the other comments (e.g., residual confounding and potential misclassification). The revised Limitations section is as follows (Pages 34–35, Lines 638–656 in the track changes version of the manuscript):

This study has several limitations. First, this was an observational study conducted in a single Japanese municipality. Therefore, the analyses do not prove causation between severe COVID-19 and the study outcomes, and our findings may have limited generalizability. Second, COVID-19 severity was inferred solely from claims data, potentially leading to misclassifications due to coding errors or incomplete claims. These misclassifications may also have affected the identification of comorbidities. In addition, the claims data did not include detailed clinical information, such as laboratory data (e.g., C-reactive protein) and severity markers (e.g., oxygen saturation and APACHE II scores). The absence of these variables may have led to residual confounding, potentially distorting our results. Third, we could not account for variations in clinical management across hospitals. Fourth, medical expenditures for comorbidities that were present before the index hospitalization were not evaluated. Finally, our analysis of expenditures excluded patients who died within 180 days post-discharge, which may have introduced survivorship bias. Although a sensitivity analysis confirmed the robustness of our results, we could not account for death as a competing risk for cost estimation. To build upon our findings, future research should aim to address these limitations. Specifically, prospective multi-center cohort studies incorporating detailed clinical data and patient-reported outcomes would provide a more comprehensive understanding of the long-term trajectory of severe COVID-19 survivors.

**------------------------------------------------------------------------------------------------------------------- Conclusion:**

***Comment #13***

*Revised Conclusion:
COVID-19 patients requiring mechanical ventilation or ECMO experience high post-discharge mortality and incur substantial medical expenditures. Comorbidities such as dementia, cancer, kidney disease, and cerebrovascular disease contribute to these adverse outcomes, underscoring the need for targeted post-discharge care and resource allocation.*

**Response to Comment #13:**

Thank you for the final suggestion regarding the Conclusion.

We agree that your proposed revision provides a more powerful and concise summary of our findings and their clinical implications—namely, the need for targeted post-discharge care and resource allocation. We have adopted this excellent suggestion, and are grateful for all of the advice (Page 35, Lines 663–666 in the track changes version of the manuscript):

COVID-19 patients requiring MV or ECMO experience high post-discharge mortality and incur substantial medical expenditures. Comorbidities such as dementia, cancer, kidney disease, and cerebrovascular disease contribute to these adverse outcomes, underscoring the need for targeted post-discharge care and resource allocation.
